# Half‐Body Radiation Therapy Results in a Prolonged Progression‐Free Interval in Canine High‐Grade Lymphoma After First Remission

**DOI:** 10.1111/vco.13050

**Published:** 2025-03-15

**Authors:** Yen‐Hao Erik Lai, Sarah Lyles, Mark Mitchell, Jayme Looper

**Affiliations:** ^1^ Department of Veterinary Clinical Sciences School of Veterinary Medicine, Louisiana State University Baton Rouge Louisiana USA; ^2^ Department of Clinical Sciences College of Veterinary Medicine, North Carolina State University Raleigh North Carolina USA; ^3^ MedVet New Orleans New Orleans Louisiana USA

**Keywords:** canine lymphoma treatment, case–control study, chemotherapy responders, combination therapy, lymphoma radiation, novel half body radiation therapy protocol

## Abstract

An optimal protocol of adding wide‐field irradiation to multi‐agent chemotherapy for dogs with lymphoma has not been established. The aim of this retrospective case–control study was to evaluate the efficacy of a protocol combining chemotherapy and half‐body irradiation (HBI) for dogs with high‐grade lymphoma. Dogs in the treatment group received cranial HBI 2 weeks after completing the second cycle of the multi‐agent chemotherapy protocol. The radiation therapy protocol consisted of 4 Gy/fraction once per day for 2 consecutive days for the cranial half body, followed by the same protocol for the caudal half 2 weeks later. The control group only received multi‐agent chemotherapy. All patients were required to have cytological confirmation of high‐grade lymphoma and achieve complete remission after two cycles of multi‐agent chemotherapy. Fourteen patients receiving HBI and 11 patients in the control group were included. The median progression‐free interval (PFI) in the HBI group (1143 days) was significantly longer than that in the control group (316 days, *p* = 0.004). In the HBI group, dogs with T cell lymphoma had statistically shorter PFI (292 days) than dogs with B cell lymphoma (2127 days, *p* = 0.0013). The median survival time in the HBI group (1924 days) was significantly longer than that in the chemotherapy‐only group (566 days, *p* = 0.0077). The predictive factors for longer PFI and ST were found in the patients who received HBI and chemotherapy (*p* = 0.0062 and 0.0252, respectively). For chemotherapy‐responding patients that completed a multi‐agent protocol, HBI significantly prolonged the time to tumour relapse compared with the chemotherapy‐only group.

## 1 Introduction

Lymphoma is one of the most prevalent malignancies in dogs, and the treatment of choice is multiagent chemotherapy. CHOP‐based multiagent chemotherapy protocols, comprised of prednisone, vincristine, cyclophosphamide and doxorubicin, with or without L‐asparaginase, have been associated with the longest remission rates and survival times (STs). This multiagent chemotherapy regimen yields an objective response rate between 80% and 98% [1, 2, 3, 4, 5, 6]. For multicentric high‐grade B cell lymphoma, the median ST spans from 12 to 18 months, with a 2‐year survival rate estimated at approximately 25% [5, 7]. Conversely, high‐grade T cell lymphoma generally exhibits a median ST between 6 and 9 months [5, 8, 9]. Prognostic indicators for peripheral nodal lymphoma are focused on immunophenotype (favouring longer survival in B cell lymphoma compared to T cell lymphoma), World Health Organisation (WHO) substage (notably, substage ‘a’ patients exhibit longer survival compared to substage ‘b’ or clinically unwell patients) and extended survival associated with an indolent subclassification [10, 11, 12, 13]. Dogs with stage I or II also generally exhibit more favourable treatment outcomes compared to those with advanced stage disease (stage III or higher).

Research using radiation therapy to treat lymphoma in dogs dates to the 1960s [14]. Two‐megavoltage (MV) whole‐body irradiation was employed, delivering a total dose ranging from 200 to 1300 R (equivalent to 1.8 to 11.4 Gy) and with a daily prescription of 0.2–0.4 Gy. However, severe adverse effects were noted in some cases, including significant bone marrow suppression and widespread bleeding secondary to thrombocytopenia in one dog. Despite these challenges, 7 out of 14 dogs exhibited partial or complete responses to radiation therapy, with STs spanning from 0.5 to 10.5 months [14]. The concept of half‐body radiation (HBI) therapy emerged in the 1980s [15]. This approach involved prescribing 7 Gy to the cranial half of the body and an equivalent dose to the caudal half, with a separation of 4–6 weeks between treatments. However, early attempts faced setbacks because of inappropriate therapeutic radiation doses and frequency. Consequently, severe side effects such as bone marrow necrosis, tumour lysis syndrome and secondary haemorrhagic gastroenteritis were observed [15]. In that study, the STs for patients with partial and complete responses were 2 and 4 months, respectively.

Larger‐scale investigations in the 2000's combining HBI with chemotherapy were shown to be safe [16, 17]. This approach involved administering a dose of 6 or 8 Gy of radiation to both the cranial and caudal halves of the body after two cycles of the CHOP protocol. Side effects primarily consisted of transient vomiting and diarrhoea and mild bone marrow suppression. The median ST of 10–18 months was comparable to historical data for patients receiving chemotherapy alone. Additional study using a low dose rate (8–14 cGy/min) for HBI, with radiation administered 2 weeks apart to the cranial and caudal regions, resulted in an improved median ST of 22 months, accompanied by 1‐, 2‐ and 3‐year survival rates of 66%, 47% and 44%, respectively [18]. It is noteworthy that the patients who received radiation therapy were primarily those who had responded well to chemotherapy, achieving complete remission (CR) following initial treatment. This finding suggests that HBI may be most effective in patients already in CR, helping clinicians better select suitable candidates. However, it remains unclear whether the long‐term survival of these patients is attributable to radiation therapy, or simply due to their strong response to chemotherapy. A recent study comparing lymphoma patients treated with multi‐agent chemotherapy to those who also received radiation therapy failed to demonstrate a clear benefit from radiation therapy due to the inclusion of poor responders in the chemotherapy‐only group [19]. Furthermore, even though the studies suggested there was improvement in the first remission duration and overall STs, an optimal protocol that improves outcomes while minimising toxicity has not been established.

The aim of this study was to evaluate a refined combination chemotherapy and HBI protocol for dogs with B or T cell high‐grade lymphoma. The second aim of this study was to assess whether incorporating the refined HBI protocol is justified in patients that responded positively to chemotherapy. In this retrospective analysis, we compared canine patients with high‐grade lymphoma who responded to multiagent chemotherapy, examining outcomes between those treated with or without our refined HBI protocol. The authors' hypotheses were that HBI would significantly prolong time to tumour relapse and survival in chemotherapy‐responding patients that completed a multi‐agent protocol compared to patients only receiving chemotherapy.

## 2 Materials and Methods

### 2.1 Study Design

A retrospective, non‐blinded, case–control study was done using clinical records from Louisiana State University from January 2014 to April 2024. The inclusion criteria for the study objects were (1) confirmed diagnosis of intermediate to large cell lymphoma based on cytology or histopathology; (2) patients with multicentric, mediastinal or hepatosplenic lymphoma were included, and patients with multicentric lymphoma were at least stage III (lymph nodes are enlarged on both sides of the diaphragm) and (3) the patients were in CR after two cycles of multi‐agent chemotherapy to proceed to HBI. The treatment response was categorised according to the Veterinary Cooperative Oncology Group (VCOG) response evaluation criteria for peripheral nodal lymphoma in dogs (v1.0) [20]. The multi‐agent chemotherapy included the CHOP‐based protocol (prednisone, vincristine, cyclophosphamide and doxorubicin) or the MOPP protocol (mechlorethamine, vincristine, procarbazine and prednisone), with or without L‐asparaginase. Flow cytometry, blood smear clinical pathology review, thoracic radiographs or abdominal ultrasound were not required for inclusion. This retrospective study included client‐owned dogs, with informed consent obtained at admission and care provided that followed good clinical practice standards.

### 2.2 Diagnosis and Staging

Clinical information obtained from the records of the study objects included age, sex, neuter status, breed, body weight and presenting signs of illness on physical exam. All patients were categorised as substage a (no clinical signs) or b (clinical signs, including gastrointestinal or respiratory signs, hypercalcemia or fever) according to the WHO system. Data retrieved from complete cell counts (CBC) and serum biochemistry profiles were used for the initial staging workup. Ionised calcium was not evaluated in all cases and was therefore not included in the analysis. Diagnostic imaging data, including thoracic radiographs and abdominal ultrasonography at diagnosis, were also collected if available.

### 2.3 Treatment Protocol

Treatment data including the date of diagnosis and treatment, induction and rescue chemotherapy protocols and the response to treatment were obtained. The induction protocol was determined by the attending medical oncologist. For the CHOP‐based protocol, the starting dose of vincristine was 0.7 mg/m [2] IV, cyclophosphamide 200–250 mg/m [2] PO, doxorubicin 30 mg/m^2^ IV and prednisone 40 mg/m^2^ PO, and the treatment duration was 25 weeks for a total of four cycles [6, 12]. The MOPP protocol and doses were described in previously published studies [21].

The HBI protocol included both cranial and caudal HBI. The cranial HBI was performed 2 weeks after the second cycle of chemotherapy. Restaging was performed prior to cranial HBI, including lymph node measurement and cytology if the lymph nodes were enlarged. Thoracic radiographs or abdominal ultrasound were only performed for patients who had lung, mediastinal lymphadenopathy or abdominal involvement at diagnosis, and cytology was only conducted when imaging findings appeared abnormal. The radiation therapy protocol entailed 4 Gy per fraction once per day for 2 consecutive days (total 8 Gy) for the cranial half body. The same dose prescription was performed on the caudal half body 2 weeks after the cranial HBI. L‐asparaginase was administered 1 week after cranial HBI, and a CBC was not required. CBCs were performed 2 weeks after cranial HBI and before caudal HBI. Patients treated with HBI continued the chemotherapy protocol after radiation therapy until the completion of treatment. Chemotherapy continued 2 weeks after caudal HBI. At 2 weeks post HBI completion, if patients had neutrophil (< 1800/μL) or platelet counts (< 50 000/μL‐grade 3 thrombocytopenia) that were too low to continue chemotherapy, L‐asparaginase would be prescribed and the chemotherapy protocol delayed for one more week. The treatment scheme is shown as Table 1.

**TABLE 1:** The treatment scheme of lymphoma patients administered with CHOP chemotherapy protocol and half‐body irradiation.

Treatment	Week																		
	1	2	3	4	6	7	8	9	11	12	13	15	17	19	21	23	25	27	29
Vincristine (0.7 mg/m^2^ IV)	•		•		•		•					•		•		•		•	
Cyclophosphamide (200–250 mg/m^2^ PO)		•				•							•				•		
Doxorubicin (30 mg/m^2^ IV)				•				•							•				•
Cr HBI									•										
L‐asparaginase (400 IU/kg SQ)										•									
Cd HBI											•								
Prednisone	2 mg/kg PO q24 h for 7days, then 1.5 mg/kg PO q24 h for 7days, then 1.0 mg/kg PO q24 h for 7days, then 0.5 mg/kg PO q24 h for 7days, then stop																		

Radiation therapy was delivered using a manual point‐dose calculation with a linear accelerator (21EX, Varian Medical Systems Inc., Palo Alto, CA) that delivered 6MV photons with a dose rate of 100 monitor units per minute at the isocenter (39–105 cGy/min at prescription point). The patients were positioned in lateral recumbency for treatment. The head and limbs in the treatment field were secured to the body with tape and wedge sponges for positioning (Figure S1). No bolus or blocks were used. Parallel opposed fields were used to deliver a homogeneous dose to the entire PTV (cranial or caudal half of the body), with the xiphoid process of the sternum serving as the landmark dividing the cranial and caudal body [16]. All treatments were recorded and verified in Aria (version 13.7). The dose was manually calculated at the midline with a source‐to‐axis distance of 100 cm. When the field size was over the limitation of the collimator (40 × 40 cm), the distance from the source was increased so that the half body could fit into the treatment field. The inverse square factor was introduced to the calculation formula to correct the dose rate. The gap of skin between the adjacent fields of the cranial and caudal half bodies was not calculated.

To match the age in the HBI group (median age of 4 years old), we included patients aged ≤ 6 years in the control group. The selection of a rescue protocol for cases of tumour relapse was determined on a case‐by‐case basis by board‐certified medical oncologists.

### 2.4 Adverse Events Related to Chemotherapy and Radiation Therapy

A CBC was obtained regularly prior to and 1–2 weeks after chemotherapy. The grading of the toxicity was defined according to VCOG common terminology criteria for adverse events (CTCAE v2) from 1 to 5 [22]. The clinical records, CBCs and serum biochemistry results were evaluated retrospectively for evidence of AEs (i.e., myelosuppression, gastrointestinal toxicity, renal toxicity or hepatotoxicity). Normal tissue toxicity potentially associated with radiation therapy was evaluated based on medical record review according to the Veterinary Radiation Therapy Oncology Group (VRTOG v2.0) [23].

### 2.5 Statistical Analysis

The distributions of the continuous data were evaluated using the Shapiro–Wilk test. The Mann–Whitney *U*‐test and Student's *t*‐test was used to compare the differences for non‐normally and normally distributed continuous data, respectively. Categorical data, including tumour stage and sex, were analysed using the Fisher's exact test or Chi‐square test. An unpaired *t*‐test was used to measure differences in the body weight between patients with B and T cell lymphoma in the HBI group. The progression‐free interval (PFI) was defined as the time from initial treatment (first‐line chemotherapy) to time of first event, including either tumour relapse or death. The patients were censored if there were no signs of tumour relapse at the time of data analysis or for cases lost to follow‐up. The tumour relapse was defined as enlarged lymph nodes or liver (for patients with liver involvement at diagnosis) and progression of clinical signs related to lymphoma with confirmation by cytology. The ST was defined as the time from initial treatment to the patients' death. The patients were censored if they were lost to follow‐up. The Kaplan–Meier survival curves for PFI and ST were generated, and the median PFI and MST between HBI and control groups were compared by log‐rank analysis. Within the HBI group, the association of immunophenotype (B‐ or T‐cell) with median PFI and MST was also evaluated by log‐rank test (T cell lymphoma was present only in the HBI group). Univariate Cox proportional hazard regression models were used to evaluate the prognostic factors for PFI and ST across all dogs in the study. Variables with *p* < 0.2 in the univariate analysis were included in a subsequent multivariate Cox proportional hazard analysis. The prognostic factors that were evaluated included treatment type (HBI as part of therapy or chemotherapy only), age, sex, body weight, breed, substage (a or b) and HBI dose rate. Pearson correlation coefficient tests were used to evaluate the association between radiation dose rate and neutrophil and platelet counts. A *p* value < 0.05 was set as a statistically significant cutoff. Statistical analysis was performed using GraphPad Prism 9 software (GraphPad, La Jolla, CA) and MedCalc Statistical Software version 22.006 (MedCalc Software Ltd., Ostend, Belgium).

### 2.6 Cell Line Validation Statement

No cell line was used.

## 3 Results

### 3.1 Study Population

Twenty‐five dogs diagnosed with high‐grade lymphoma via cytology by a board‐certified clinical pathologist were included in the study. Fourteen patients had HBI as a part of their lymphoma treatment (HBI group) and 11 patients met the inclusion criteria for the chemotherapy‐only group (control group). One dog in the HBI group had confirmed lymphoblastic lymphoma cytology in the liver, a thickened gastrointestinal wall and enlarged lymph nodes in the abdomen (Table S1); however, the PCR for antigen receptor rearrangements (PARR) failed to detect clonal rearrangements of immunoglobulin or T cell receptor genes due to low cellularity. This dog was retained in the study based on cytology confirmation of large cell lymphoma. The median ages of the HBI and control groups were 4 (range from 2 to 9) and 5 (range from 3 to 6) years old, respectively. Information regarding signalment, body weight, approximate stage, substage and immunophenotype are summarised in Table S1. There were no statistically significant differences (all *p* > 0.05) for age, sex or breed between the HBI and control groups. Within the HBI group, the mean body weight of the dogs with T cell lymphoma was statistically higher (mean 37.16 kg, *p* = 0.0241) than that of dogs with B cell lymphoma (mean 22.09 kg). The mean difference ± SEM was 15.07 ± 5.76 kg.

All patients were at least stage III, and the stage distribution differed significantly between the HBI and control groups (*p* = 0.048). The number of dogs in the HBI and control groups by stage were stage III (10 vs. 4), stage IV (2 vs. 0) and stage V (2 vs. 6), respectively. However, full staging was not performed in most of the dogs in the HBI group (see staging information in Table S1). In the HBI group, one patient (7.1%, 1/14) was screened using a peripheral blood smear; 6 out of 14 (43%) HBI patients did not have thoracic or abdominal images to investigate liver, spleen or lung involvement; and one dog (7.1%, 1/14) only had a thoracic radiograph without abdominal imaging. Consequently, these patients could only be minimally confirmed to stage III. Similarly, 2 of 11 patients in the control group did not undergo full staging and were also assigned as stage III. As a result, tumour stage was not included for prognostic factor analysis. In the HBI and control groups, 10 (71.4%) and 8 (72.7%) dogs were classified as substage a, respectively, while 4 (36.3%) and 2 (18.2%) dogs were classified as substage b, respectively. The substage was not defined in the dog with rectal lymphoma in the control group. There was no statistically significant difference (*p* > 0.99) in substage between the groups.

Immunophenotype was determined by flow cytometry or PARR; PARR was used when flow cytometry was not available. A majority of the patients were diagnosed with multicentric B cell lymphoma (HBI group, 8/14 [57.1%]; control group, 8/11 [72.7%]). In the HBI group, four (28.6%) patients were diagnosed with multicentric T cell lymphoma and one (7.1%) patient had assumed T cell hepatic lymphoblastic lymphoma (as described previously, T cell was not confirmed by PARR due to low cellularity). One patient with mediastinal lymph node enlargement, confirmed as T cell lymphoma, also had generalised peripheral lymphadenopathy and was therefore classified as multicentric lymphoma rather than primary mediastinal lymphoma. One (7.1%) dog did not proceed with immunophenotyping in the HBI group. One (9.1%) patient in the control group was diagnosed as rectal B cell lymphoma, and two (18.2%) patients had no further diagnostics for immunophenotyping of T or B cell lymphoma (Table S1).

Three out of 14 (21%) patients (2 T cell lymphoma and one suspect T cell lymphoma without confirmation) in the HBI group received the MOPP protocol as the first‐line chemotherapy. The remainder of the patients in the study used CHOP‐based chemotherapy (HBI group, 11/14 [79%]; control group, 11/11 [100%]) as the initial treatment. All patients completed the initial chemotherapy protocol. Of the six (42.9%) dogs in the HBI group that relapsed during the follow‐up period, four (28.6%) dogs received rescue chemotherapy. All but two patients (9/11, 81.8%) in the control group had tumour relapse (one had rectal lymphoma and one developed osteosarcoma) and all of them received rescue chemotherapy. The rescue chemotherapy included CHOP (nine dogs, 69.2%), MOP or MOPP (two dogs, 15.4%), single‐agent doxorubicin (one dog, 7.7%) and rabacfosadine (one dog, 7.7%). The second rescue chemotherapy included MOPP, lomustine and rabacfosadine. L‐asparaginase was administered based on the clinical judgement of the attending medical oncologists.

### 3.2 Half‐Body Radiation Therapy

The dose rate calculated for the cranial HBI ranged from 39.5 to 105.8 cGy/min. The data for the cranial HBI dose rate were normally distributed (72.7 ± 20.16 cGy/min). The caudal HBI dose rate was not normally distributed among the patients; the median, 95% confidence interval and range were 95.9, 61.54–102.6 and 57.2–104.2 cGy/min, respectively.

### 3.3 Adverse Events

**FIGURE 1:**
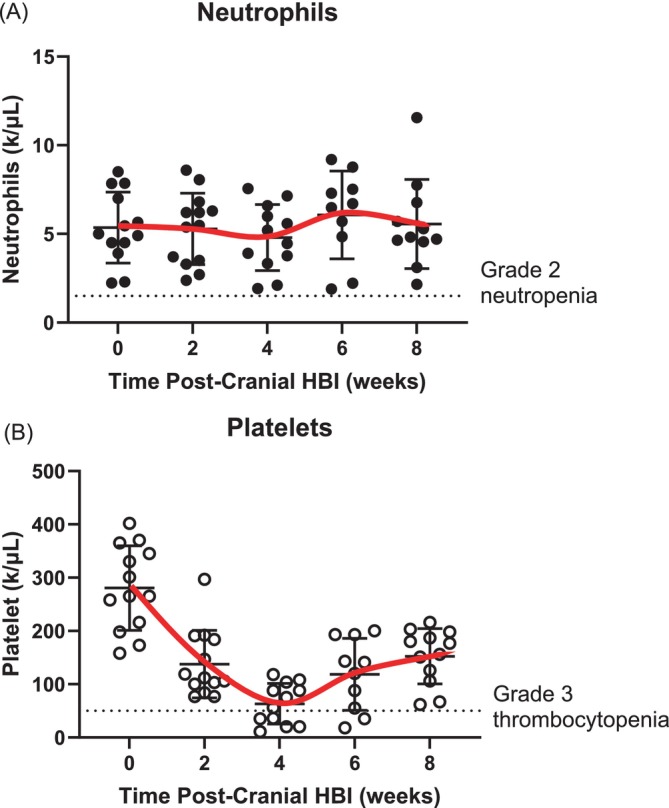
Neutrophil count (A) and platelet count (B) after cranial half body radiation. The curve represented the mean with the error bar depicting SD. The individual dots represent neutrophil or platelet count from a dog. The dashed line indicated the level of grade 2 neutropenia (1500/ μL) and grade 3 thrombocytopenia (50 k/μL).

The median time between caudal half HBI and the date of continuing chemotherapy (third cycle of chemotherapy) was 14 days. CBC data were incomplete in four patients: three due to missing medical records from the primary veterinarian and one due to data loss from a medical record system change. However, chemotherapy was approved after the CBC results were reviewed by medical oncologists via private communication, ensuring appropriate oversight despite the missing records. A CBC was not required nor obtained in all patients 1 week post‐cranial HBI. None of the patients with CBC results developed anaemia associated with HBI. Although one patient had grade 2 neutropenia 1 week post‐caudal HBI and two patients had grade 1 neutropenia 2 weeks post‐caudal HBI, all the patients had neutrophil counts > 1800/μL 2 weeks post‐caudal HBI (Table 2 and Figure 1A).

**TABLE 2:** The proportion of dogs experiencing hematologic toxicity that were treated with HBI (number of dogs/total number of dogs that have available data^a^).

Time	Toxicity	Grade 1	Grade 2	Grade 3	Grade 4
Pre‐HBI	Neutropenia	15% (2/13)	0%	0%	0%
	Thrombocytopenia	8% (1/13)	0%	0%	0%
Post‐Cr HBI 2 weeks	Neutropenia	15% (2/13)	0%	0%	0%
	Thrombocytopenia	46% (6/13)	23% (3/13)	0%	0%
Post‐Cd HBI 2 weeks	Neutropenia	17% (2/12)	0%	0%	0%
	Thrombocytopenia	25% (3/12)	33% (4/12)	17% (2/12)	25% (3/12)
Post‐Cd HBI 4 weeks	Neutropenia	10% (1/10)	0%	0%	0%
	Thrombocytopenia	30% (3/10)	20% (2/10)	10% (1/10)	10% (1/10)
Post‐Cd HBI 6 weeks	Neutropenia	8% (1/12)	0%	0%	0%
	Thrombocytopenia	17% (2/12)	17% (2/12)	0%	0%

^a^ Data were unavailable due to missing medical records from the primary veterinarian. However, chemotherapy was approved after the CBC results were reviewed by medical oncologists via private email or messages, ensuring appropriate oversight despite the missing records. CBC records were not available in one patient due to the medical record system change and data lost until week 6 post‐CdRT. CBC was not required and not obtained in all patients on post‐Cr and Cd HBI 1 week; thus, the data were not presented in the table.

Thrombocytopenia was common after HBI. One week post‐cranial HBI treatment, platelet count data were available for six patients; CBC was not required and therefore not obtained for all patients at this time. Of these, three dogs (50%) had grade 1 thrombocytopenia and two dogs (33%) had grade 2 thrombocytopenia. On the week of caudal HBI (2 weeks from cranial HBI), platelet count data were available for 13 patients. Six dogs (43%) had grade 1 thrombocytopenia and three dogs (23%) had grade 2 thrombocytopenia. Two weeks post‐caudal HBI (4 weeks from cranial HBI), three dogs (25%) had grade 1 thrombocytopenia, four dogs (33%) had grade 2 thrombocytopenia, two dogs (17%) had grade 3 thrombocytopenia and three dogs (25%) had grade 4 thrombocytopenia. Four weeks post‐caudal HBI (6 weeks from cranial HBI), three dogs (30%) had grade 1 thrombocytopenia, two dogs (20%) had grade 2 thrombocytopenia, one dog (10%) had grade 3 thrombocytopenia and one dog had grade 4 thrombocytopenia (10%). None of the dogs had grade 3 or 4 thrombocytopenia 6 weeks post‐caudal HBI. The trend of the platelet count is presented in Figure 1B. The incidence of platelet count rebound was observed in dogs with a higher dose rate. The platelet counts on weeks 6 and 8 post‐cranial HBI were positively correlated to the caudal HBI dose rate (*p* = 0.0449 and *p* < 0.0001, respectively). On week 8 post‐cranial HBI, the platelet count was also positively correlated to the mean dose rate (*p* = 0.019). All cases of thrombocytopenia appeared to be subclinical, as none of the patients exhibited abnormal bleeding, melena, hematochezia, bruising, ecchymosis or petechiae.

All the patients were prescribed anti‐nausea medications, probiotics or metronidazole as needed for gastrointestinal discomfort. Probable radiation‐induced acute gastrointestinal toxicity was reported in three (21.4%) patients. Two (HBI‐2 and HBI‐3, see Table S2) dogs had grade 2 diarrhoea 1–2 weeks post‐caudal HBI, although one (HBI‐3) of the patients had historical chronic diarrhoea. One patient (HBI‐4) had decreased appetite for about 2 weeks (grade 2) post‐caudal HBI. Alopecia and leukotrichia were reported in 5/14 (36%) and 3/14 (21%) of the irradiated patients, respectively (Table S2). Necropsy was performed on one patient in the HBI group with multicentric T‐cell lymphoma. The patient was euthanised on day 629 due to weakness of hind limbs, fever and pain on spinal palpation. The necropsy revealed stage V lymphoma with tumour cells present in multiple lymph nodes, liver, and lungs. No evidence of pathological changes related to radiation therapy was reported.

### 3.4 Response to Treatment and Prognostic Factors

**FIGURE 2:**
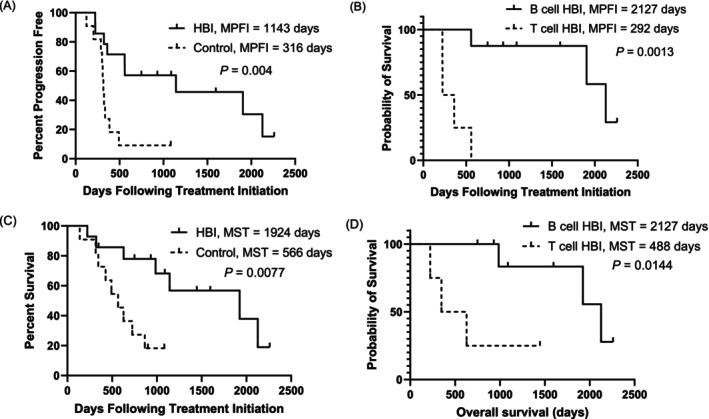
Kaplan–Meier curves of progression‐free interval (PFI) and overall survival time (ST). (A) The median PFI in HBI compared is significantly longer than that of the control group. (B) Within the HBI group, dogs with T cell lymphoma had a significantly shorter median PFI than dogs with B cell lymphoma. (C) The median ST in HBI is significantly longer than that of the control group. (D) The median ST was statistically longer in B cell lymphoma than in T cell lymphoma within the HBI group. The tick marks represent censored data. *p* values represent univariate log rank values.

Of the 14 dogs in the HBI group, 7 (50%) dogs had lymphoma relapse and 2 (14.3%) dogs developed other diseases during the follow‐up (metastatic digit melanoma and primary lung tumour with carcinomatosis, respectively) without evidence of lymphoma relapse. In the control group, all patients completed chemotherapy induction, and two (18.2%) patients did not experience tumour relapse during the follow‐up. One had rectal lymphoma and remained alive at the time of data analysis (1083 days), but the other patient developed osteosarcoma on day 316 and was euthanised. Therefore, 5 out of 14 patients (36%) in the HBI group and 1 out of 11 patients (9%) in the control group were censored for PFI analysis (Table S1). For those patients still in remission, the median follow‐up time was 1086 days (range 749–2261 days). The median PFI in the HBI group was 1143 days (range: 223–2261 days), which was significantly longer (*p* = 0.004) than in the control group (316 days, range: 123–1083 days, Figure 2A). Four dogs in the HBI group with T cell lymphoma had statistically shorter (*p* = 0.0013) PFI (292 days, range 223–559 days) than dogs with B cell lymphoma (2127 days, range 557–2261) (Figure 2B).

Five out of 14 dogs in the HBI group (35.7%) and 2 out of 11 dogs (18.2%) in the control group were alive at the time of data analysis. Two patients in the HBI group were lost to follow‐up; one was in remission at the time of the last visit (follow‐up of 1597 days) and one had relapsed lymphoma (follow‐up of 348 days). Those nine patients were censored for overall survival time analysis. The median follow‐up time for censored patients was 1088 days in the HBI group and 991 days in the control group (median follow‐up time for all censored patients was 1083 days). Two dogs in the HBI group died of other causes during follow‐up: one from metastasis of digital melanoma at 1143 days and the other from carcinomatosis of pulmonary carcinoma at 2127 days. In the control group, one dog developed osteosarcoma and was euthanised. All remaining patients died from lymphoma.

The median ST in the HBI group was 1924 days (range 223–2261 days), which was significantly longer (*p* = 0.008) than the control group (566 days, range 138–1083 days, Figure 2C). Four dogs in the HBI group with T cell lymphoma had a median survival time of 488 days (range 223–1447 days), and the survival time was significantly shorter (*p* = 0.014) than that of dogs with B cell lymphoma (2127 days, range 321 to 2261 days, Figure 2D). In the HBI group, the 1‐ and 2‐year survival rates were 79% and 71%, respectively. The three‐year survival rate was 43% (6 out of 14). However, three patients had not yet reached 3 years of follow‐up and were still alive; therefore, the three‐year survival rate is expected to be higher. The follow‐up time for these three patients who had not reached 3 years was 749, 931, and 1088 days. In the control group, the 1‐ and 2‐year survival rates were 73% and 27%, respectively. Two patients remained alive at the time of analysis and had survival times of 899 (multicentric, unknown immunophenotype lymphoma) and 1083 days (rectal B cell lymphoma).

Age, body weight, sex, substage, treatment group, rescue chemotherapy and dose rate were included for prognostic factor analysis in all dogs in the HBI and control groups. By using univariate Cox regression analysis for PFI, the prognostic factors that were associated with shorter PFI included increased body weight (*p* = 0.02), control treatment group (*p* = 0.004) and potentially younger age (*p* = 0.1688). The factors that were associated with shorter ST included increased body weight (*p* = 0.0025), without chemotherapy rescue (*p* = 0.026), substage b (*p* = 0.021) and control group (*p* = 0.0178). The factors were further analysed with a multivariate Cox regression model, which showed the prognostic factors for shorter PFI and ST were the patients receiving chemotherapy only (*p* = 0.0062 and 0.0252, respectively) and increased body weight (*p* = 0.0178 and 0.0081, respectively). We further evaluated the prognostic factor within the HBI group, but the increased body weight did not predict shorter PFI or ST. When evaluating the correlation between body weight and dose rate, body weight was inversely correlated with cranial HBI dose (*p* = 0.012) rate and mean dose rate (*p* = 0.001). However, within the HBI group, the radiation dose rate did not predict the survival outcome.

## 4 Discussion

This study demonstrated that our HBI protocol was both effective and well tolerated for canine lymphoma patients in clinical remission after two cycles of multi‐agent chemotherapy. Our findings supported our hypothesis that in chemotherapy‐responsive patients, HBI prolongs the time to tumour relapse and improves survival compared to those only receiving multi‐agent chemotherapy. Moreover, half‐body radiation therapy was safe for canine lymphoma patients, with the major side effects being subclinical neutropenia and thrombocytopenia.

The overall survival time and 1‐ and 2‐year survival rates in this study were higher than the previously published study [18]. This difference may be due to variations in the treatment protocol (8 Gy versus 6 Gy), dose rate, smaller study population or the relatively younger median age of the dogs in our study. We included younger dogs in the control group to match the HBI group, aiming to minimise population variation. According to the literature, age was only considered a prognostic factor for survival in 20% of the lymphoma studies [24]. For example, one early study identified age younger than 7 years as a negative prognostic factor [25]. However, the fact that T‐cell lymphoma tends to present in younger animals can be a confounding factor.

We used 8 Gy treated over 2 consecutive days (4 Gy per fraction) for cranial and caudal HBI. The determination of total dose and fractionation was based on previously published protocols [17, 26]. Regarding dose rate, 267 cGy/min was reported in Gustafon's study, while the dose rate was 8–14 cGy in Lurie's study [17, 18]. Two out of six dogs (33%) had grade 4 neutropenia and grade 4 diarrhoea or vomiting when using a dose rate of 267 cGy/min. In comparison, when using a low dose rate of 8–14 cGy to deliver 4 Gy in each fraction, none of the patients had grade 4 bone marrow toxicity. Given this information, the dose rate of 8–14 cGy/min was expected to take 30–50 min to complete one fraction of radiation therapy. Prolonged anaesthesia time was considered a potential risk factor for complications and possible prolonged recovery time [27, 28]; however, this was not investigated in the patients treated with radiation therapy. Although the goal of a low dose rate was to decrease the toxicity to normal tissues, it may not have a clinically significant impact on toxicity and could possibly decrease the efficacy of cancer cell control compared with a higher dose rate [29]. A study looking at the bone marrow toxicity in total body irradiation in dogs showed a fractionated protocol significantly reduced the toxicity in bone marrow with a 60 cGy/min dose rate compared to single dose radiation when applying 3 Gy total body irradiation [30]. Although the dose, dose rate and fractionation were different and we were not using total body irradiation, our findings indicate that a fractionated HBI protocol with dose rates up to 104 cGy/min did not cause significant clinical toxicity. The present study also cannot correlate the bone marrow toxicity and the dose rate. With a higher dose rate, each fraction can be completed without the prolonged anaesthesia time, and all of the patients tolerated the protocol well with medical support in our study. However, further studies related to the dose rate and fractionation should be investigated to characterise the radiation toxicity. Interestingly, the platelet count showed a positive correlation with dose rate at weeks 6 and 8. It remains unclear whether this statistical significance reflects a type I error or if increased radiation dose rates promote platelet production. There is no known literature describing a correlation between dose rate and platelet counts in HBI. A larger sample size would be needed to assess repeatability and investigate the underlying mechanism.

Multi‐agent chemotherapy is widely used as a first‐line treatment for canine high‐grade lymphoma. The current study incorporated HBI during the chemotherapy protocol, demonstrating prolonged PFI compared to the patients only receiving chemotherapy. This finding is consistent with the previously reported HBI studies [18, 19]. The rationale of administering radiation therapy after two cycles of multi‐agent chemotherapy is to minimise the cancer cells in the body, thereby achieving the best outcome for residual circulating cancer cell sterilisation. However, this inclusion criterion introduces a selection bias because it excludes patients who did not respond to chemotherapy. Although the factor for complete response to the first induction of chemotherapy has not been reported to be associated with long‐term survival, it is generally believed that patients who failed in the first multi‐agent chemotherapy have more aggressive, chemoresistant lymphoma. Therefore, we included a control group cohort that achieved a complete response after two cycles of multi‐agent chemotherapy (week 11). In the present study, the MST for patients receiving chemotherapy alone was 566 days, which is longer than the reported MST in B cell multicentric lymphoma [6, 31]. Theoretically, chemotherapy induction followed by radiation therapy should destroy residual lymphoblastic cells, minimising the number of residual cancer cells in patients receiving chemotherapy. Our results suggest that those patients who respond well to chemotherapy derive additional benefit from including HBI as part of their treatment regimen. Additionally, four dogs with T cell lymphoma in the HBI group had a median survival time of 488 days, which is longer than the generally reported 6–9 months in T cell lymphoma patients [8, 9]. Although none of the control group patients had T cell lymphoma, preventing direct comparison, it is possible that HBI provides additional benefit for patients with T cell lymphoma.

The prognostic factor evaluation showed that patients not receiving HBI and at increased body weight were more likely to have shorter PFI and ST. However, the increased body weight was no longer a predictive factor for the HBI group. When comparing PFI and ST between B cell and T cell lymphoma in the HBI group, T cell lymphoma dogs had statistically shorter PFI than B cell lymphoma dogs, and the MST trended shorter in dogs with T cell lymphoma. Therefore, because the dogs with T cell lymphoma in the present study had higher mean body weights, the immunophenotype was the confounding factor that predicted the outcome, not the body weight.

Indeed, due to the selection criteria, we do not know if the patients who do not achieve a complete response after two cycles of chemotherapy would benefit from HBI. It is possible that without achieving a complete response, the residual tumour volume may be too high for HBI to effectively control the disease. This is particularly relevant because we spaced out the cranial and caudal HBI by 2 weeks. This interval allows cancer cells to potentially regenerate in the caudal half of the body while we are irradiating the cranial half. If the tumour volume remains high (e.g., palpable enlarged lymph nodes) after two cycles of chemotherapy, the cancer cells may redistribute during this 2‐week window, escaping irradiation of the caudal half when it is treated later. Using total body radiation therapy at one time may significantly increase the bone marrow toxicity, necessitating a reduction in radiation dose and/or hospitalisation for monitoring. This reduction would likely decrease the efficacy of tumour cell eradication. Therefore, while our current approach shows promise, the specific impact of HBI on patients who do not achieve a complete response with initial chemotherapy remains uncertain and warrants further investigation. In the present study, patients with naïve high‐grade lymphoma were included, while patients that had relapsed lymphoma were excluded. One study has investigated total body irradiation therapy in dogs with relapsed lymphoma; however, there remains minimal evidence supporting the effectiveness of HBI in relapsed cases of multicentric lymphoma in dogs [32].

The rationale for irradiating the cranial half body before the caudal half body is to reduce the risk of GI side effects, which could interrupt radiation therapy if the caudal half is treated first. However, the GI side effects were generally manageable, with only 21% of the patients treated with HBI reporting such side effects. This low prevalence should be interpreted cautiously because it may have been influenced by incomplete medical records, patients responding well to supportive medication or recovery by the time they continued chemotherapy. Since GI toxicity was low, it might be feasible to perform caudal HBI before cranial HBI for patients with hepatic or splenic lymphoma, or those with enlarged lymph nodes primarily in the abdominal cavity. Further study to determine whether the order of the HBI therapy (cranial vs. caudal half body radiation) impacts outcome is warranted.

The present study has inherent retrospective limitations. Ideally, weekly CBCs would be done to closely monitor post‐radiation toxicity. However, the timing of the CBC rechecks in this retrospective study depended upon individual patient clinical presentations and clinician judgement, with most patients having blood counts every other week when continuing their chemotherapy. Furthermore, as with most veterinary studies, increasing the sample size could provide more information regarding prognostic factors such as stage, substage and different types or locations of lymphoma. The staging was incomplete in most of the patients in the HBI group, with only a single peripheral blood smear pathology review performed. Without a blood smear pathology review or bone marrow sample, some stage III or stage IV lymphoma may have actually been downstaged. A prospective study enrolling patients with complete staging is needed to provide more insights into potential differences in outcomes between stages III, IV and V in the HBI and control groups. Regardless, a previous report found no significant difference in survival between stages III, IV and V lymphoma [33]. Additionally, ionised calcium concentration levels were not investigated in the present study, which may or may not correlate with treatment outcomes.

In conclusion, HBI was effective and well tolerated for canine lymphoma patients in clinical remission after two cycles of multi‐agent chemotherapy. For patients responding to chemotherapy, our HBI protocol combined with multi‐agent chemotherapy significantly prolonged the time to tumour relapse and increased survival time compared to the chemotherapy‐only group.

## Ethics Statement

This retrospective study included client‐owned dogs, with informed consent obtained at admission. Patient care was provided in accordance with good clinical practice standards. Ethical approval was not required by the lead author's institution for a retrospective study in which no research was conducted on live animals.

## Conflicts of Interest

The authors declare no conflicts of interest.

## Supporting information


Data S1.

## Data Availability

The data that support the findings of this study are available on request from the corresponding author. The data are not publicly available due to privacy or ethical restrictions.
