# Supplementary material for: Half‐Body Radiation Therapy Results in a Prolonged Progression‐Free Interval in Canine High‐Grade Lymphoma After First Remission
Source: Vet Comp Oncol. 2025 Mar 15;23(2):236–45. doi: 10.1111/vco.13050 (PMC12082781; doi:10.1111/vco.13050)
Supplement: Supplementary file 1 — Data S1. [file VCO-23-236-s001.docx]

| 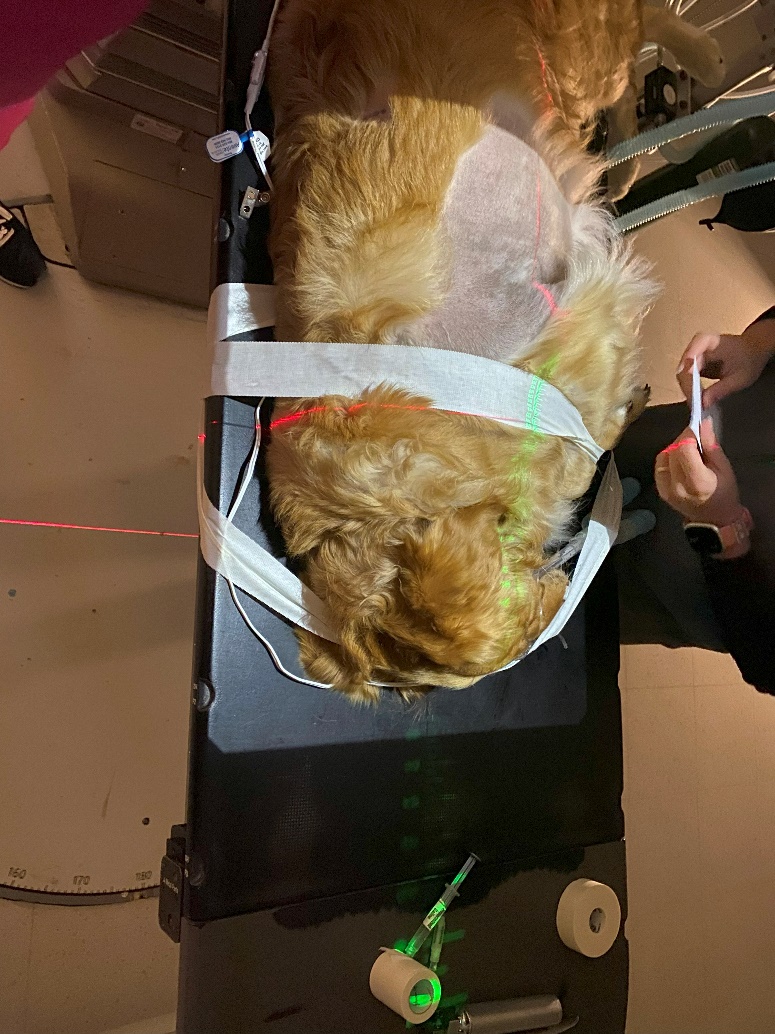 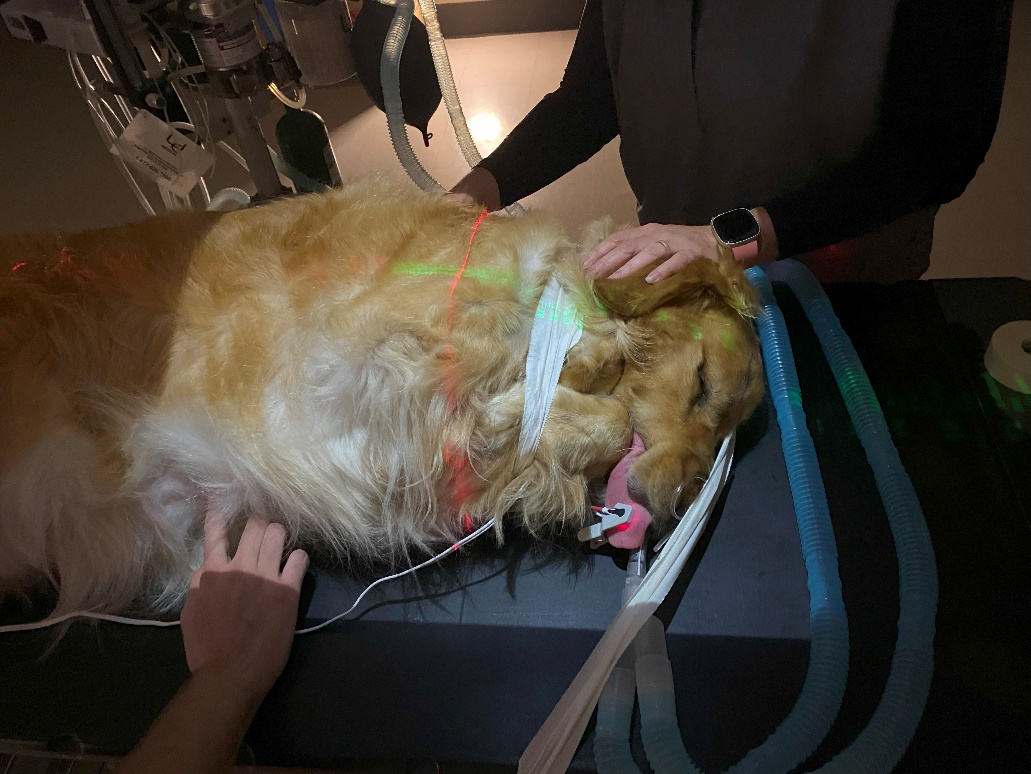 |
| --- |
| **Supplementary Figure 1.** Cranial half-body irradiation in a patient with lateral recumbency. The head and limbs were secured to the body with tape to ensure homogeneous radiation dose delivery to the cranial half. The xyphoid process of the sternum was used as the anatomical landmark to distinguish between the cranial and caudal halves. For caudal half-body irradiation, a similar lateral recumbency setup was used, with the hind limbs and tail securely taped to the body. |

**Supplementary Table 1.** The demographics of the patients in the study.

| Study ID | Age | Sex | Breed | BW (kg) | Staging information | Stage | Substage | IPT | PFI | ST |
| --- | --- | --- | --- | --- | --- | --- | --- | --- | --- | --- |
| HBI-1 | 4 | MC | Mastiff mix | 51 | Incomplete (no PB smear path review, CXR, or AUS) | 3^§^ | a | T | 223 | 223 |
| HBI-2 | 4 | MC | German Shepherd | 36.6 | Complete | 3 | a | T | 224 | 348^†^ |
| HBI-3 | 2 | MC | Bull terrier | 24.4 | Incomplete (no PB smear path review, CT chest and abdomen WNL) | 3^§^ | a | B | 749^†^ | 749^†^ |
| HBI-4 | 8 | FS | Miniature Schnauzer | 5.65 | Incomplete (no PB smear path review or AUS; CXR- WNL) | 3^§^ | a | B | 931^†^ | 931^†^ |
| HBI-5 | 4 | MC | Corgi | 13.6 | Incomplete (no PB smear path review or AUS; CXR- diffuse pulmonary involvement) | 5^§^ | b | B | 1088^†^ | 1088^†^ |
| HBI-6 | 4 | MC | Golden Retriever | 32.2 | Incomplete (no PB smear path review or AUS; CXR- cranial mediastinal/sternal lymphadenopathy) | 4^§^ | b | T | 360 | 629 |
| HBI-7 | 2.5 | FS | German shorthaired pointer | 28.8 | Incomplete (no PB smear path review, CXR, or AUS) | 3^§^ | a | n/a | 321 | 321 |
| HBI-8 | 4 | FS | Rhodesian ridgeback | 25.8 | Incomplete (no PB smear path review, CXR, or AUS) | 3^§^ | a | B | 557 | 985 |
| HBI-9 | 2.5 | FS | Dalmatian | 23.6 | Incomplete (no PB smear path review, CXR, or AUS) | 3^§^ | a | T | 559 | 1447^†^ |
| HBI-10 | 9 | MC | Labrador Retriever | 42.4 | Incomplete (no PB smear path review or CXR; AUS- enlarged liver, spleen, gastric thickening and enlarged abdominal LNs) | 5^§^ | b | n/a^¶^ | 1143 | 1143 |
| HBI-11 | 6 | FS | Mixed | 24.4 | Incomplete (no PB smear path review; CXR- WNL; AUS- enlarged and mottled spleen with enlarged abdominal LNs) | 4^§^ | b | B | 2261^†^ | 2261^†^ |
| HBI-12 | 8 | FS | Mixed | 16.7 | Incomplete (no PB smear path review, CXR, or AUS) | 3^§^ | a | B | 1597^†^ | 1597^†^ |
| HBI-13 | 6 | FS | Mixed | 27.7 | Incomplete (no PB smear path review, CXR, or AUS) | 3^§^ | a | B | 2127 | 2127 |
| HBI-14 | 5 | MC | Golden Retriever | 38.5 | Incomplete (no PB smear path review, CXR, or AUS) | 3^§^ | a | B | 1905 | 1924 |
| Control-1 | 6 | MC | Chihuahua | 3.07 | Complete | 5 | b | n/a | 307 | 899^†^ |
| Control-2 | 6 | FS | English Pointer | 20.6 | Complete | 5 | a | B | 493 | 493 |
| Control-3 | 6 | FS | Pitbull | 24.4 | Complete | 3 | a | B | 385 | 865 |
| Control-4 | 6 | MC | Doberman Pinscher | 51 | Incomplete (PB smear path review- WNL; no CXR or AUS) | 3^§^ | a | B | 316 | 316 |
| Control-5 | 5 | MC | English Bull dog | 34.8 | Incomplete (PB smear path review- WNL; no CXR or AUS) | 3^§^ | a | n/a | 123 | 138 |
| Control-6 | 5 | FS | Golden retriever | 34.8 | Complete | 5 | a | B | 323 | 724 |
| Control-7 | 4 | MC | Poodle mix | 41.6 | Complete | 5 | a | B | 336 | 627 |
| Control-8 | 4 | MC | Mininature Schnauzer | 8.6 | Complete; rectal lymphoma | n/a | n/a | B | 1083^†^ | 1083^†^ |
| Control-9 | 4 | MC | Pitbull | 37.6 | Complete | 3 | a | B | 287 | 427 |
| Control-10 | 4 | FS | Schnauzer mix | 12.6 | Complete | 5 | a | B | 300 | 566 |
| Control-11 | 3 | FS | Golden retriever | 30.4 | Complete | 5 | b | B | 203 | 345 |

Abbreviations: MC, male castrated, FS, female spayed; BW, body weight; CXR, chest x-rays (thoracic radiographs); AUS, abdominal ultrasound; PB, peripheral blood; n/a, not performed/available; WNL, within normal limits; IPT, immunophenotype; LN, lymph node.

^§^ Patients without complete staging (thoracic or abdominal image, and peripheral blood smear pathology review).

^¶^ Lymphoblastic lymphoma was diagnosed by the cytology of liver and gastric thickening, as well as the enlarged lymph nodes in the abdomen. Additional sampling of liver aspirations sent to PARR had low cellularity and T cell immunophenotype cannot be confirmed.

^†^ Censored data.

**Supplementary Table 2.** The dose rate and adverse event (AE) of individual patients in HBI group.

| Study ID | Dose rate (cranial) | Dose rate (caudal) | Dose rate (mean) | Bone marrow AE^§^ | Gastrointestinal AE | Skin AE | Rescue chemotherapy |
| --- | --- | --- | --- | --- | --- | --- | --- |
| HBI-1 | 54.94505 | 74.34944 | 64.64725 | Grade 4 thrombocytopenia |  |  | no |
| HBI-2 | 54.34783 | 93.45794 | 73.90289 | Grade 1 neutropenia;  Grade 3 thrombocytopenia | Grade 2- intermittent diarrhea within 1-2 weeks post caudal HBI |  | yes |
| HBI-3 | 74.62687 | 97.08738 | 85.85712 | Grade 2 thrombocytopenia | Grade 2- intermittent diarrhea within 1-2 weeks post caudal HBI | Grade 1- alopecia | n/a |
| HBI-4 | 105.2632 | 104.1667 | 104.7149 | Grade 4 thrombocytopenia | Grade 2- decreased appetite post caudal HBI for 2 weeks | Grade 1- alopecia | n/a |
| HBI-5 | 105.8201 | 102.5641 | 104.1921 | Grade 3 thrombocytopenia |  | Grade 1- alopecia | n/a |
| HBI-6 | 58.47953 | 67.79661 | 63.13807 | Grade 1 neutropenia;  Grade 4 thrombocytopenia |  | Grade 1- leukotrichia | yes |
| HBI-7 | 69.68641 | 99.50249 | 84.59445 | Grade 2 thrombocytopenia |  | Grade 1- alopecia | no |
| HBI-8 | 78.43137 | 97.56098 | 87.99617 | Grade 2 thrombocytopenia |  |  | yes |
| HBI-9 | 88.49558 | 57.30659 | 72.90108 | Grade 2 thrombocytopenia |  |  | n/a |
| HBI-10 | 64.51613 | 61.53846 | 63.0273 | Grade 2 neutropenia;  Grade 2 thrombocytopenia |  | Grade 1- leukotrichia | n/a |
| HBI-11 | 39.52569 |  | 39.52569 | Grade 1 neutropenia;  Grade 3 thrombocytopenia |  | Grade 1- leukotrichia | n/a |
| HBI-12 |  |  |  | Grade 1 thrombocytopenia |  | Grade 1- alopecia | n/a |
| HBI-13 |  |  |  | Grade 3 thrombocytopenia |  |  | n/a |
| HBI-14 |  |  |  |  |  |  | yes |

^§^ Most severe event within 8 weeks post cranial HBI.
